# The synthetic steroid tibolone exerts sex-specific regulation of astrocyte phagocytosis under basal conditions and after an inflammatory challenge

**DOI:** 10.1186/s12974-020-1719-6

**Published:** 2020-01-28

**Authors:** Andrea Crespo-Castrillo, Luis-Miguel Garcia-Segura, Maria-Angeles Arevalo

**Affiliations:** 1 Instituto Cajal, Consejo Superior de Investigaciones Científicas (CSIC), Madrid, Spain; 2 Centro de Investigación Biomédica en Red Fragilidad y Envejecimiento Saludables (CIBERFES), Instituto de Salud Carlos III, Madrid, Spain

**Keywords:** Astrocytes, Phagocytosis, Tibolone, Estrogen receptor, Androgen receptor, LPS, Sex differences, Cellular debris

## Abstract

**Background:**

Tibolone is a synthetic steroid used in clinical practice for the treatment of climacteric symptoms and osteoporosis. Active metabolites of tibolone, generated in target tissues, have an affinity for estrogen and androgen receptors. Astrocytes are direct targets for estrogenic compounds and previous studies have shown that tibolone protects brain cortical neurons in association with a reduction in reactive astrogliosis in a mouse model of traumatic brain injury. Since phagocytosis is a crucial component of the neuroprotective function exerted by astrocytes, in the present study, we have assessed whether tibolone regulates phagocytosis in primary astrocytes incubated with brain-derived cellular debris.

**Methods:**

Male and female astrocyte cell cultures were obtained from newborn (P0-P2) female and male Wistar rats. Astrocytic phagocytosis was first characterized using carboxylate beads, *Escherichia coli* particles, or brain-derived cellular debris. Then, the effect of tibolone on the phagocytosis of Cy3-conjugated cellular debris was quantified by measuring the intensity of Cy3 dye-emitted fluorescence in a given GFAP immunoreactive area. Before the phagocytosis assays, astrocytes were incubated with tibolone in the presence or absence of estrogen or androgen receptor antagonists or an inhibitor of the enzyme that synthesizes estradiol. The effect of tibolone on phagocytosis was analyzed under basal conditions and after inflammatory stimulation with lipopolysaccharide.

**Results:**

Tibolone stimulated phagocytosis of brain-derived cellular debris by male and female astrocytes, with the effect being more pronounced in females. The effect of tibolone in female astrocytes was blocked by a selective estrogen receptor β antagonist and by an androgen receptor antagonist. None of these antagonists affected tibolone-induced phagocytosis in male astrocytes. In addition, the inhibition of estradiol synthesis in the cultures enhanced the stimulatory effect of tibolone on phagocytosis in male astrocytes but blocked the effect of the steroid in female cells under basal conditions. However, after inflammatory stimulation, the inhibition of estradiol synthesis highly potentiated the stimulation of phagocytosis by tibolone, particularly in female astrocytes.

**Conclusions:**

Tibolone exerts sex-specific regulation of phagocytosis in astrocytes of both sexes, both under basal conditions and after inflammatory stimulation.

## Background

Tibolone is a synthetic steroid that is currently used in clinical practice for the treatment of climacteric symptoms and to prevent osteoporosis in postmenopausal women [1, 2]. In addition to some reports suggesting that tibolone may improve semantic memory and cognition in postmenopausal women [3–6], neuroprotective actions of the steroid have been reported in different animal and cellular models. Thus, tibolone improves cognition deficits and decreases CA1 dendritic spine pruning caused by ovariectomy in female rats [7, 8] and in male rats exposed to ozone improves memory and protects the hippocampus from oxidative stress and cholinergic alterations [9, 10].

In vitro studies have shown that tibolone exerts protective actions on glial cell lines. Thus, the steroid reduces inflammation and prevents oxidative damage in BV-2 microglia exposed to palmitic acid [11] and protects T98G cells from glucose deprivation and palmitic acid toxicity [12–14], reducing oxidative stress and preserving mitochondrial membrane potential [14, 15]. Furthermore, tibolone decreases in vivo the reactive response of microglia and astrocytes after a stab wound lesion of the cerebral cortex in ovariectomized female mice [16].

Tibolone is metabolized in the liver and other tissues, including the brain, in three active steroids: 3α- and 3β-hydroxytibolone and Δ4-tibolone. 3α- and 3β-hydroxytibolone have an affinity for estrogen receptors (ERs), while Δ4-tibolone binds to androgen and progesterone receptors [17, 18]. Previous studies have shown that tibolone 3-hydroxy metabolites activate ERs in human primary astrocytes [19] and that ERs, mainly ERβ, mediate different actions of tibolone on T98G astrocyte-like cells [14, 15]. The direct actions of tibolone on astrocytes and astrocyte-like cells through ERs, together with the decreased neuronal loss in association with a reduction of reactive astrogliosis in the injured cortex of tibolone-treated mice [16], suggest that astrocytes may be involved in the neuroprotective actions of the steroid. Indeed, it is known that astrocytes mediate the neuroprotective actions of estradiol and other ER ligands in different CNS (central nervous system) pathologies [20, 21].

Phagocytosis is a crucial component of the control exerted by astrocytes to maintain CNS homeostasis. It contributes to physiological neuronal and synaptic pruning during development and adulthood [22–27] and to the remodeling of myelin and the clearance of cellular debris under pathological conditions [23, 26, 28, 29]. Phagocytosis is, in fact, essential for the homeostatic function of astrocytes and a decreased phagocytic activity is one of the characteristics of those reactive astrocytes that lose their neuroprotective function [30].

Regulation of microglia phagocytosis is known to contribute to neuroprotective estrogenic actions [31–33]. However, it is unknown if astrocyte phagocytosis is also regulated by estrogenic compounds. Thus, in the present study, we have assessed whether tibolone regulates phagocytosis in astrocytes, using primary mouse astroglia cultures incubated with cellular debris derived from hippocampus. Since astrocytes show different molecular and morphological sex differences [34–40], our study was conducted on cultures obtained separately from male and female animals. The findings indicate that tibolone enhances phagocytosis in primary astrocytes under resting and inflammatory conditions. The effect of tibolone on phagocytosis involves different molecular mechanisms in male and female astrocytes and it is modulated by the endogenous balance of testosterone and estradiol.

## Methods

### Animals

Wistar rats from the Institute Cajal colony were used for this study. Animals were housed under controlled temperature (22 ± 2 °C) and light (12-h light/dark cycle) conditions and with food and water available ad libitum. Animals procedures followed the European Parliament and Council Directive (2010/63/EU) and the Spanish regulation (R.D. 53/2013 and Ley 6/2013, 11th June) on the protection of animals for experimental use and were approved by our institutional animal use and care committee (Comité de Ética de Experimentación Animal del Instituto Cajal) and by the Consejeria del Medio Ambiente y Territorio (Comunidad de Madrid, PROEX 134/17).

### Astrocyte and microglia cultures

Primary cultures of forebrain astrocytes were obtained from newborn (P0) to 2-day-old (P2) male and female pups. Animals were sexed via measurement of anogenital distance, the brain was extracted, meninges were removed and the cerebral cortex was isolated under a dissected microscope. Dissociated cortical cells from male or female pups were plated separately in 75-cm^2^ flasks, coated with 10 μg/ml poly-l-lysine and grown at 37 °C and 5% CO_2_ in Dulbecco’s Modified Eagle’s Medium (DMEM, ThermoFisher Scientific, Madrid) supplemented with 10% heat-inactivated fetal bovine serum (FBS), 10% heat-inactivated horse serum, and antibiotic-antimycotic (DMEM 10:10:1) [41]. After reaching confluence, cells were shaken at 280 rpm for 16 h at 37 °C. The supernatant was collected for preparation of microglia cultures and cells that remained attached to the flask were kept in the incubator for the later isolation of astrocytes. For microglia cultures, the supernatant was centrifuged for 10 min at 280 g. Purified microglia were plated at a density of 25,000 cells/cm^2^ on poly-l-lysine (10 μg/ml)-coated glass coverslips and maintained at 37 °C and 5% CO_2_ in phenol red-free and antibiotic-free RPMI medium (GIBCO, ThermoFisher Scientific), supplemented with 1% heat-inactivated FBS. For astrocyte cultures, cells that remained attached to the flask were treated for 20 min at 37 °C with 10% trypsin-EDTA. Then, detached cells were centrifuged for 10 min at 280 g. Purified astrocytes were plated at a density of 10,000 cells/cm^2^ on poly-l-lysine (10 μg/ml)-coated glass coverslips and maintained at 37 °C and 5% CO_2_ in phenol red-free and antibiotic-free DMEM medium (GIBCO), supplemented with 1% heat-inactivated FBS, sodium pyruvate (1 mM, GIBCO), and L-glutamine (10 mM, GIBCO).

### Pharmacological treatments

Astrocytes were incubated for 6 h in DMEM with 10% FBS and then 15 h with the following test compounds, either alone or in combination in DMEM 0.1% FBS: tibolone (100 nM; Sigma-Aldrich); ERα antagonist 1,3-Bis(4-hydroxyphenyl)-4-methyl-5-[4-(2-piperidyletoxy) phenol]-1H-pyrazole (MPP, 100 nM; Sigma-Aldrich); the ERβ antagonist 4-[2-phenyl-5,7-bis (trifluoromethyl) pyrazolo [1,5-a] pyrimidin-3-yl] phenol (PHTPP, 100 nM; Tocris, Bristol); the G-protein-coupled ER (GPER) antagonist (3aS,4R,9bR)-4-(6-bromo-1,3-benzodioxol-5-yl)-3,4,5,9B-tetrahydro-3H-cyclopenta[c]quinolone (G15, 100 nM; Tocris); the androgen receptor antagonist flutamide (100 nM; Tocris); or the aromatase inhibitor letrozole (100 nM; Tocris). Twenty-four hours before phagocytosis assay astrocytes were incubated with DMEM serum-free medium with the previous compounds plus lipopolysaccharide (LPS) isotype O26:B6 (1 μg/ml; Sigma-Aldrich, Tres Cantos, Madrid). The concentrations used for the test compounds were chosen on the basis of previous studies [11, 42]. After the incubation with the test compounds, phagocytosis activity was assessed as indicated below.

### Preparation of Cy3 conjugated brain-derived cellular debris

Cellular debris was obtained from male and female mouse embryonic day 17 hippocampi. The hippocampi were dissected out and dissociated to single cells after digestion for 15 min with 0.5% of trypsin (Worthington Biochemicals, Freehold, NJ) and DNase I (Sigma-Aldrich). Cells were then centrifuged at 280 g rpm for 5 min and the pellet was resuspended in 1 ml of Neurobasal culture medium (Invitrogen, ThermoFisher Scientific). The cell suspension was sonicated at 20 kHz for 2 s and the cellular debris were labeled using Cy™3 (Mono-Reactive Dye Pack; Amersham Biosciences, VWR, Radnor, PA) according to the manufacturer’s instructions. Cy3 conjugated cellular debris was stored at 4 °C until their use.

### Phagocytosis assays

The phagocytosis activity of primary astrocytes or microglia was assessed using carboxylate beads, *Escherichia coli* (*E*. *coli*) particles or brain-derived cellular debris to mimic unspecific phagocytosis, phagocytosis activated by bacterial pathogens or phagocytosis activated by neural cell signals, respectively. Thus, primary astrocyte or microglia cultures were incubated for 1 h at 37 °C with either 1 μl/ml Fluoresbrite© yellow green (YG) Carboxylate Microspheres (1.00 μm; Polysciences, Inc., Warminster, PA), pH-sensitive pHrodo™ Green *E*. *coli* BioParticles™ Conjugate (2 μl/ml; Fisher Scientific) or mouse Cy3-conjugated brain-derived cellular debris (10 μl/ml). In the latter case, male and female astrocyte or microglia cultures were incubated with cellular debris obtained from male and female hippocampi, respectively. For the negative control group, astrocyte culture was placed on ice and then the pre-cooled cell debris was added and incubated on ice for 1 h. Cell cultures were observed in a Leica DMI6000 fluorescent microscope equipped with a Leica DFC350 FX digital camera and with a Leica TCS SP5 direct confocal microscope. Astrocytes and microglial cellular profiles were recognized by phase-contrast microscopy. In addition, astrocytes for quantitative image analysis were recognized by anti-glial fibrillary acidic protein (GFAP) labeling (see next sections).

### Immunocytochemistry

After the incubation with fluorescent beads, *E*. *coli* particles or brain-derived cellular debris, astrocyte cultures were washed twice with phosphate-buffered saline (PBS) and fixed for 20 min at room temperature with 4% paraformaldehyde in PBS. After several washes with PBS-gelatin and permeabilized with PBS-triton x-100, astrocyte cultures were incubated with rabbit GFAP protein antibody (diluted 1:1000; DAKO, Agilent, Santa Clara, CA), followed by incubation with a goat anti-rabbit Alexa488-conjugated secondary antibody (1:1000; Jackson Immuno-Research Europe Ltd., Ely, Cambridgeshire). After washing with PBS, glass coverslips were mounted on slides with Vectashield antifade mounting medium containing DAPI (Vector Laboratories, Burlingame, CA).

### Image analysis

Internalization of fluorescent beads, *E*. *coli* particles and cellular debris within the cytoplasm of GFAP immunoreactive cells was confirmed by assessing yellow green, pHrodo™ Green and Cy3 dye-emitted fluorescence, respectively, in Z-stack images that were visualized and photographed on a Leica TCS-SP5 confocal system. Then, image analysis of the astrocyte cultures was performed on microphotographs obtained on a Leica DMI6000 fluorescent microscope using a × 20 objective and a Leica DFC350 FX digital camera. All microphotographs for quantitative analysis were taken with the same intensity and exposure. Fluorescence intensity was assessed using the Fiji imaging processing package (ImageJ 1.52n, National Institutes of Health, USA). The amount of cellular internalization of Cy3-conjugated cellular debris was quantified by measuring the intensity of Cy3 dye-emitted fluorescence in a given GFAP immunoreactive area. Cell images were obtained from at least 4 independent cultures for each experimental group. Ten random images were obtained for each culture and experimental condition and between 150 and 1000 cells were analyzed per experimental group.

### Statistical analysis

Data are presented as median ± ranges, since the values obtained in the phagocytosis assays did not follow a normal distribution. The ranges included the highest and lowest values of intensity, including both phagocytic and non-phagocytic cells in the analysis. Statistical analyses were performed using GraphPad Prism software version 5.0 for Windows. Differences between two experimental groups were analyzed by Mann-Whitney *U* test. To compare three or more groups, the Kruskal-Wallis test, followed by the post hoc Dunn’s test, was used. Differences were considered statistically significant at *p* < 0.05. The *n* for statistical analysis was the number of cells (*n* = 150–1000 cells per experimental group). Differences between proportion of phagocytic and non-phagocytic cells were studied using Fisher’s exact test.

## Results

### Characterization of the phagocytic activity of primary astrocytes

**Fig. 1:**
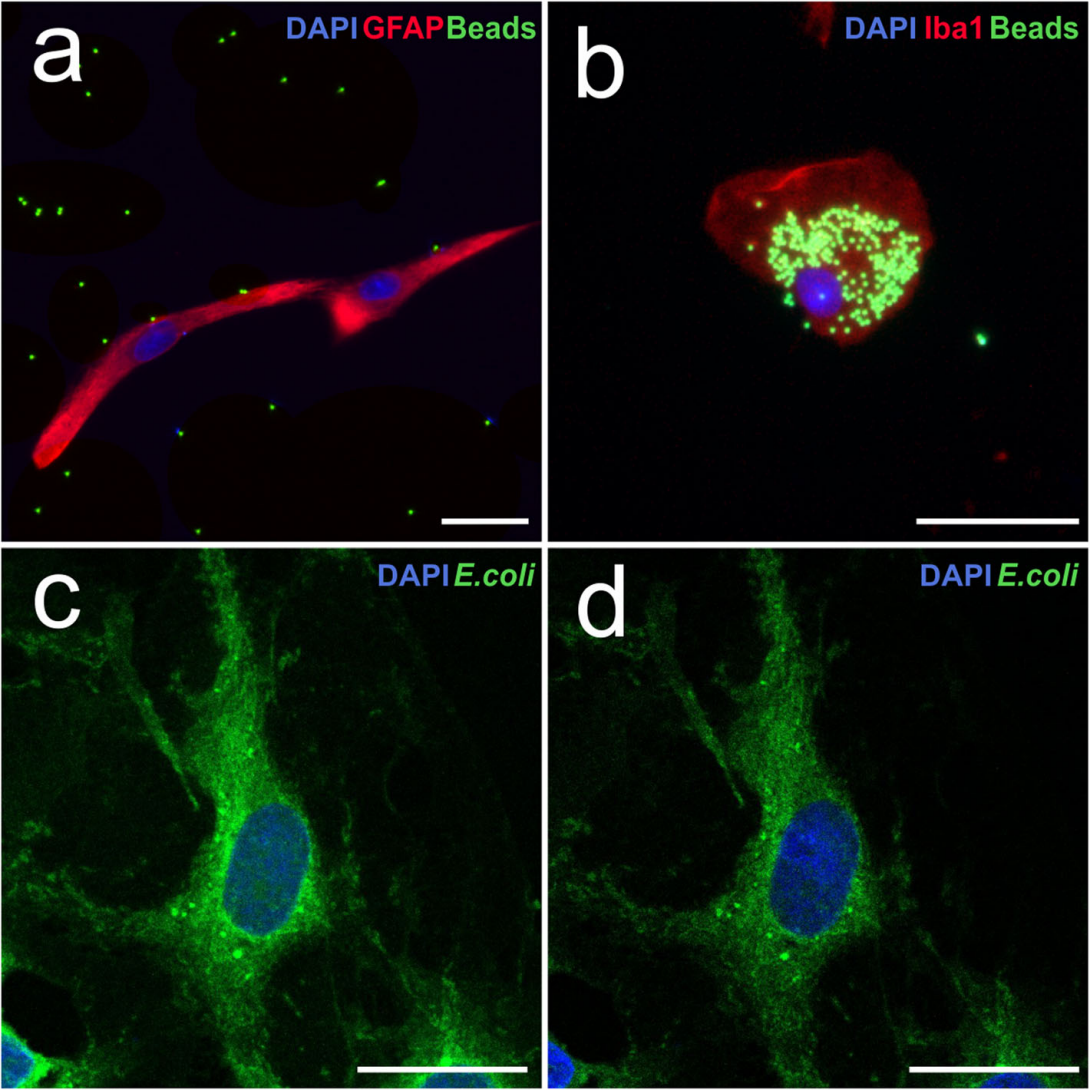
Phagocytosis of fluorescent beads and *E*. *coli* particles. **a** Fluorescence microscopic image of an astrocyte marked with anti-GFAP antibody (red) after incubation with fluorescent beads (green). **b** Fluorescence microscopic image of a cell of microglia marked with anti-Iba1 antibody (red) after incubation with fluorescent beads (green). **c** Confocal projection image of an astrocyte after *E*. *coli* (green) phagocytosis. **d** Z-stack image of the same astrocyte. The dotted line delineates the limits of the cell cytosol. Cell nuclei were stained with DAPI. Scale bar 25 μm

Astrocyte cultures were incubated for 1 h with fluorescent beads, *E*. *coli* particles or brain-derived cellular debris to evaluate its phagocytic activity. Microglial cell cultures, incubated under the same conditions, were used as positive controls. Figure 1 a shows a representative Z-stack image of the phagocytosis of fluorescent beads by an astrocyte. As shown in the figure, the number of beads engulfed by astrocytes was almost zero. In contrast, numerous internalized fluorescent beads were observed in microglia (Fig. 1b).

**Fig. 2:**
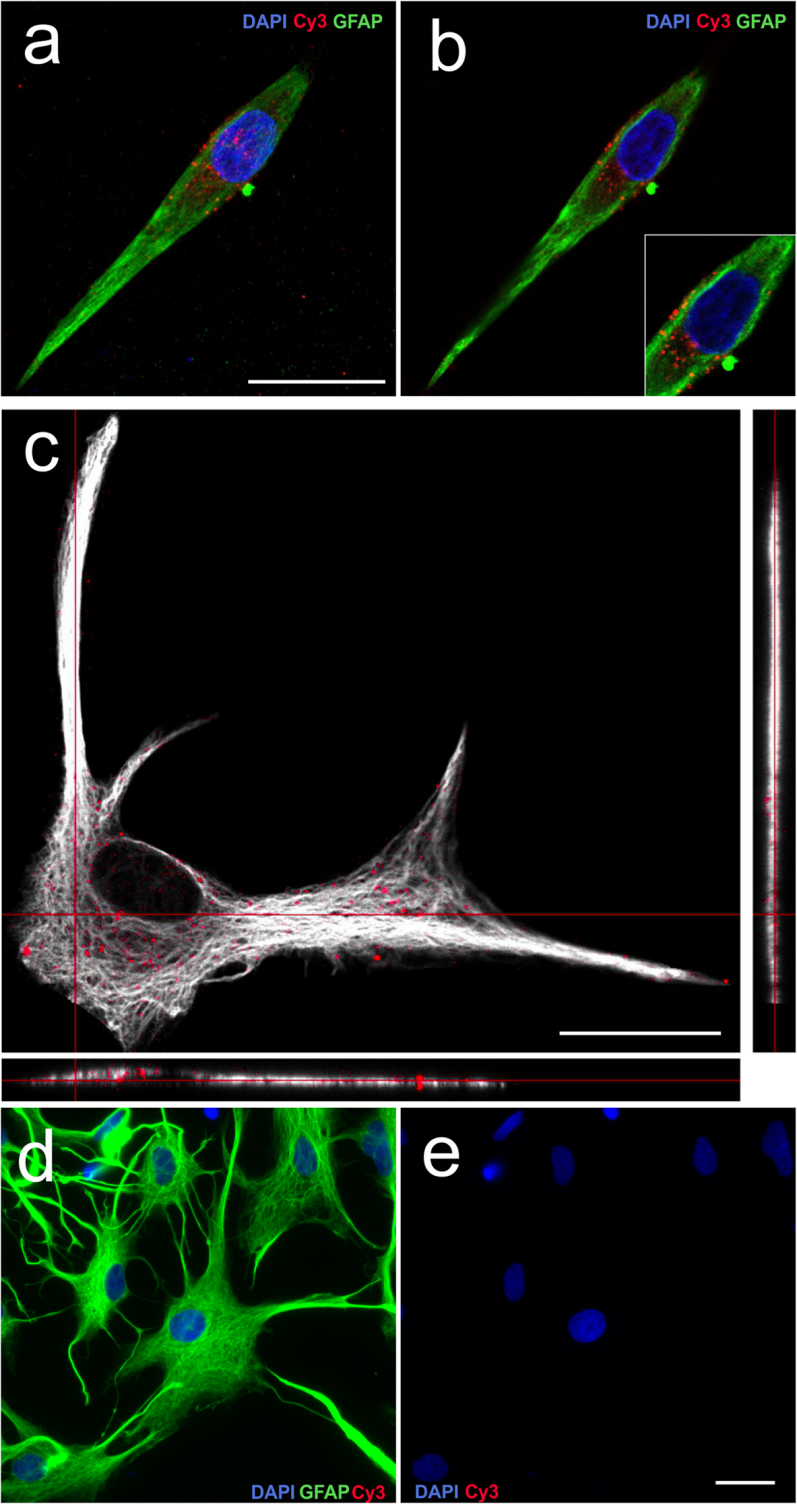
Phagocytosis of brain-derived cellular debris. **a** Confocal projection image of a GFAP (green) immunostained astrocyte after the phagocytosis of cellular debris (red). **b** Z-stack image of the same astrocyte. **c** Confocal image in *xy*, *xz*, and *yz* planes of an astrocyte after the phagocytosis of cellular debris. **d** Negative control of astrocytic phagocytosis of cellular debris at 4 °C marked with anti-GFAP antibody (green), Cy3 (red), and DAPI (blue). **e** Negative control of astrocytic phagocytosis of cellular debris at 4 °C marked with Cy3 (red) and DAPI (blue). Scale bar 25 μm

Figure 1 c and d show representative examples of the phagocytosis of *E*. *coli* particles by astrocytes in a projection and in a Z-stack image, respectively. In contrast to the low phagocytosis of fluorescent beads, numerous bacterial particles were phagocyted by astrocytes. Astrocytes were also able to efficiently phagocyte brain-derived cellular debris, as illustrated by Fig. 2 a (projection image), b (Z-stack image), and c (image in *xy*, *xz*, and *yz* planes). Microglial cells were also able to phagocytose numerous bacterial particles and cellular debris (not shown). Astrocytic phagocytosis was performed in a specific manner due to negative control was not able to phagocytose cellular debris at 4 °C (Fig. 2d, e).

From these experiments, we may conclude that, at least under our culture conditions, primary astrocytes were able to internalize *E*. *coli* particles and cellular debris, but they did not internalize carboxylated beads as efficiently as primary microglia. After the confirmation that astrocyte phagocytic activity can be studied in monolayer cultures, only the phagocytosis of brain-derived cellular debris was assessed in the following experiments.

### Tibolone stimulates the phagocytosis of brain-derived cellular debris by male and female astrocytes

**Fig. 3:**
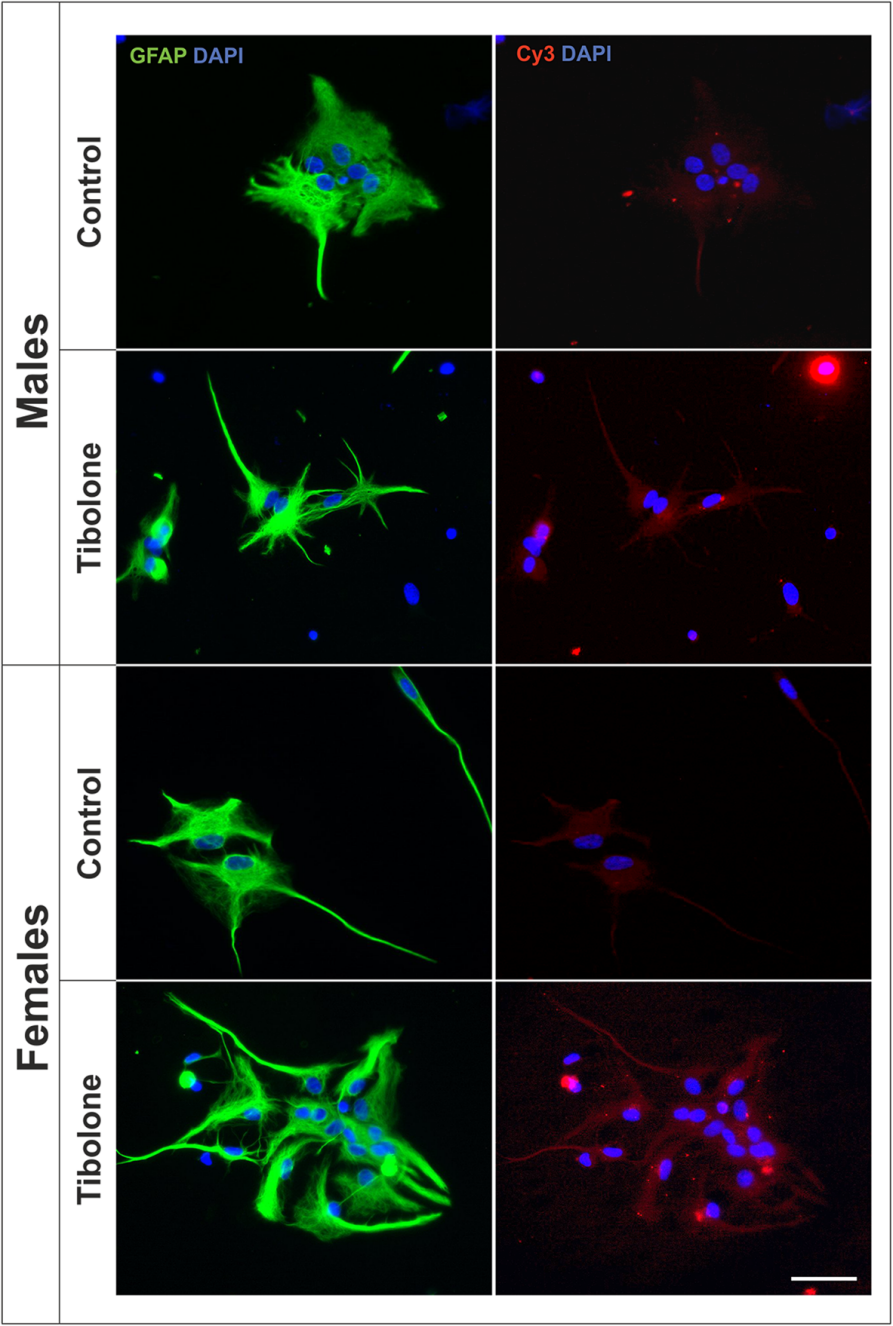
Tibolone stimulates phagocytosis of brain-derived cellular debris in male and female astrocytes. Representative confocal images of male and female astrocyte cultures treated for 39 h with tibolone or control medium and then incubated for 1 h with Cy3-conjugated brain-derived cellular debris (red) and immunostained for GFAP (green). Cell nuclei were stained with DAPI. Scale bar 50 μm

Figure 3 shows representative images of the internalization of Cy3-conjugated cellular debris by male and female astrocytes under control conditions and after the treatment for 39 h with tibolone. As shown in the figure, intracellular Cy3 labeling (red fluorescence signal) was similar in male and female astrocytes under basal conditions and was more intense in the astrocytes treated with tibolone than in control astrocytes.

**Fig. 4:**
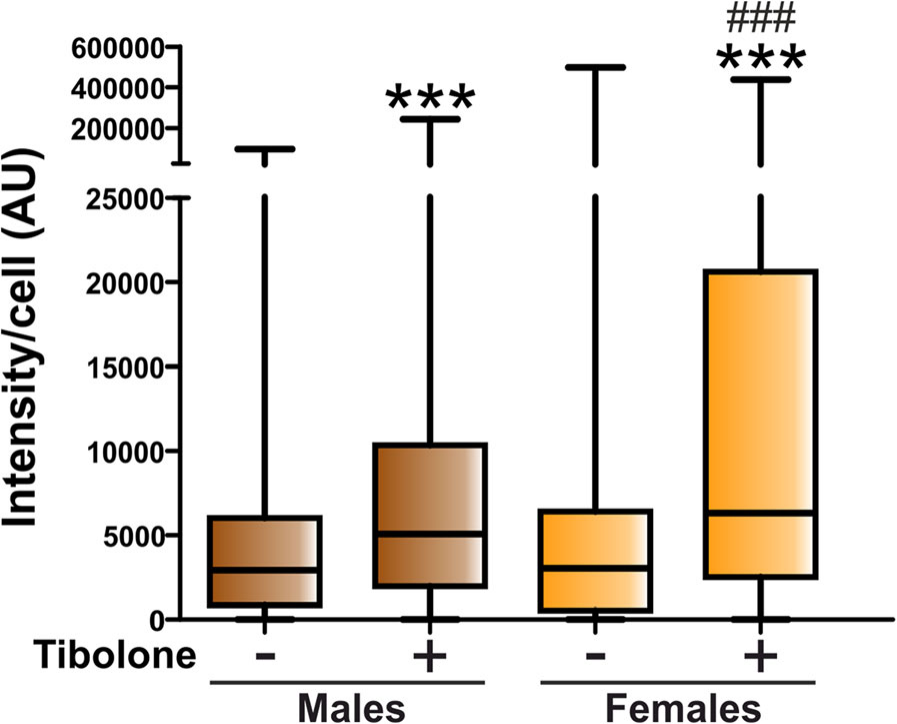
Quantitative analysis of the effect of tibolone on the phagocytosis of brain-derived cellular debris. Cy3 fluorescence intensity per cell in male and female astrocytes treated for 39 h with tibolone or control medium and then incubated for 1 h with Cy3-conjugated brain-derived cellular debris. Data are presented as median ± ranges. Significant differences: ***p < 0.001 versus the control groups, ^###^*p* < 0.001 versus the male group treated with tibolone

The results of the quantitative analysis of the effect of tibolone on the internalization of cellular debris by astrocytes are shown in Fig. 4. The amount of Cy3 internalization was similar in male and female astrocytes under basal conditions. Only a small proportion of cells showed no cellular debris internalization and this proportion of non-phagocytic cells was similar in male and female cultures (4.91% and 2.87%, respectively; *p* = 0.68). Tibolone exerted a significant stimulatory effect on the internalization of Cy3 labeling in both male and female astrocytes, with the effect being significantly higher in female than in male astrocytes (Fig. 4). The stimulatory effect of tibolone on astrocyte phagocytosis was not due to a difference in the proportion of non-phagocytic cells, which was not significantly affected by the steroid (1.17% and 3.68% of tibolone-treated male and female cells, respectively; *p* = 0.62).

### Role of estrogen and androgen receptors in the regulation of phagocytosis by tibolone

Tibolone could exert its effects through different steroid receptors, including ERs and androgen receptor [17, 43]. To determine whether ERs or androgen receptor was involved in the regulation of phagocytosis by tibolone under basal conditions, astrocyte cultures were treated with selective receptor antagonists for 1 h and then with tibolone or control medium for additional 39 h. The following receptor antagonists were tested: the ERα antagonist MPP (100 nM), the ERβ antagonist PHTPP (100 nM), the GPER antagonist G15 (100 nM), and the androgen receptor antagonist flutamide (100 nM).

**Fig. 5:**
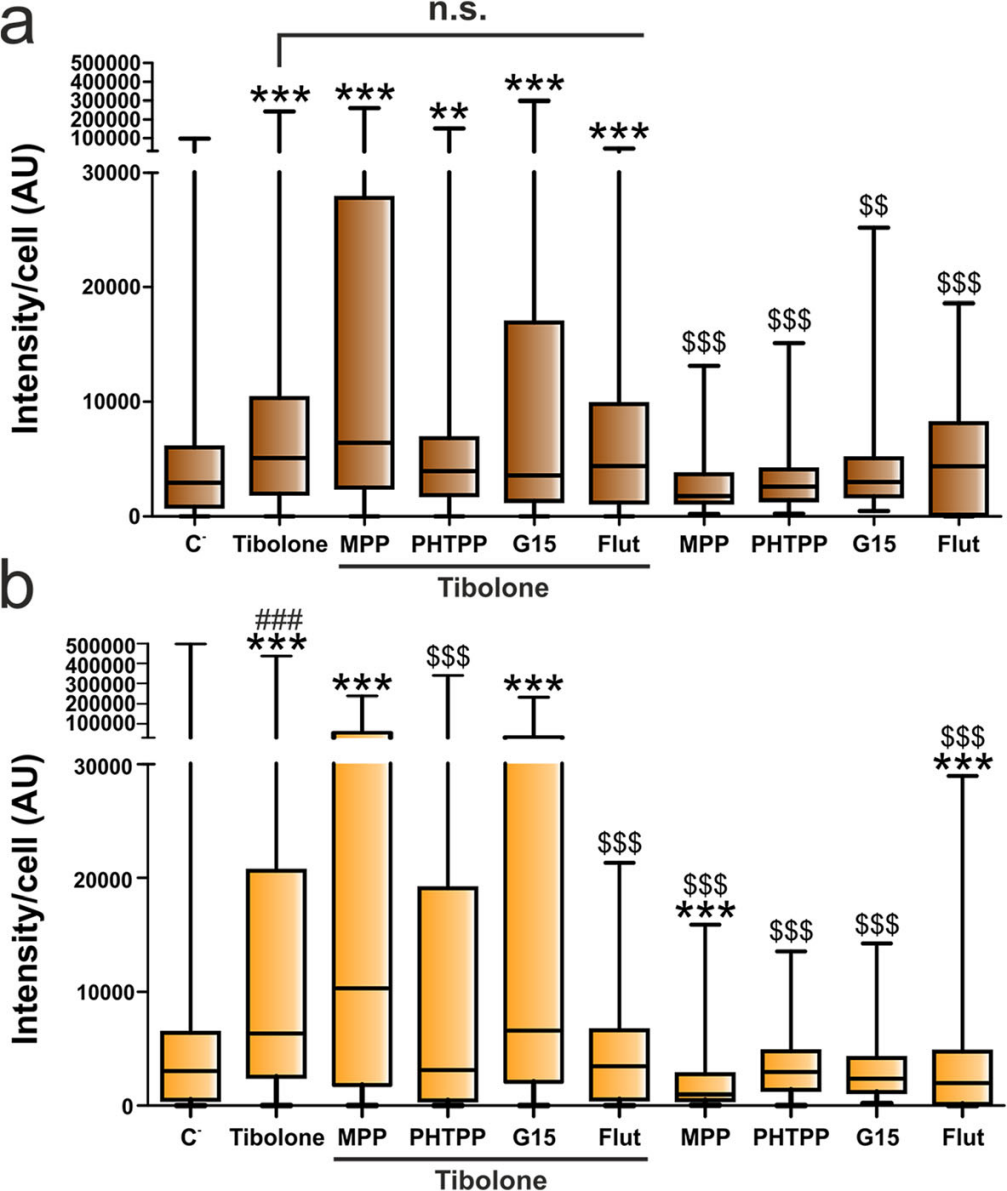
Effect of tibolone, estrogen receptor antagonists, and flutamide on the phagocytosis of brain-derived cellular debris. Cy3 fluorescence intensity per cell in male (**a**) and female (**b**) astrocytes treated for 39 h with tibolone, tibolone and the ERα antagonists MPP, tibolone and the ERβ antagonist PHTPP, tibolone and the GPER antagonist G15, tibolone and the androgen receptor antagonist flutamide, the antagonists alone, or control medium. Cells were then incubated for 1 h with Cy3-conjugated brain-derived cellular debris. Data are presented as median ± ranges. Significant differences: ***p < 0.001, **p < 0.01 versus the control group of the same sex, ^$$$^*p* < 0.001, ^$$^*p* < 0.01 versus the tibolone group of the same sex, ^###^*p* < 0.001 versus the male group treated with tibolone. n.s., non-significant; Flut, flutamide

As shown in Fig. 5 a, none of the tested receptor antagonists was able to significantly modify the effect of tibolone on Cy3 internalization by male astrocytes. In contrast, both the ERβ antagonist PHTPP and the androgen receptor antagonist flutamide blocked the effect of tibolone on the phagocytosis of cellular debris by female astrocytes (Fig. 5b). These findings indicate that the effect of tibolone on the phagocytosis of cellular debris by primary astrocytes is not a simple estrogenic effect and it involves different mechanisms in male and female cells. Furthermore, both the ERα antagonist MPP and flutamide exerted, per se, a significant inhibitory effect on the phagocytosis of cellular debris by female astrocytes, suggesting that the activation of steroid receptors by endogenous signals is involved in the regulation of phagocytosis in these cells.

### Aromatase inhibition has opposite outcomes on the regulation of phagocytosis by tibolone in male and female astrocytes

Astrocytes have the machinery to synthesize steroids, including testosterone and its metabolite estradiol, from cholesterol [44, 45]. The production of estradiol from testosterone is mediated by the enzyme aromatase, whose activity has been reported to be higher in female astrocytes [46]. The observed effects of the antagonists of ERα and androgen receptor on the basal internalization of Cy3-conjugated cellular debris suggest that the endogenous ligands of these receptors may be involved in the regulation of phagocytosis. To explore this possibility, astrocytes were incubated for 40 h with control medium or letrozole, an aromatase inhibitor, in the presence or absence of tibolone.

**Fig. 6:**
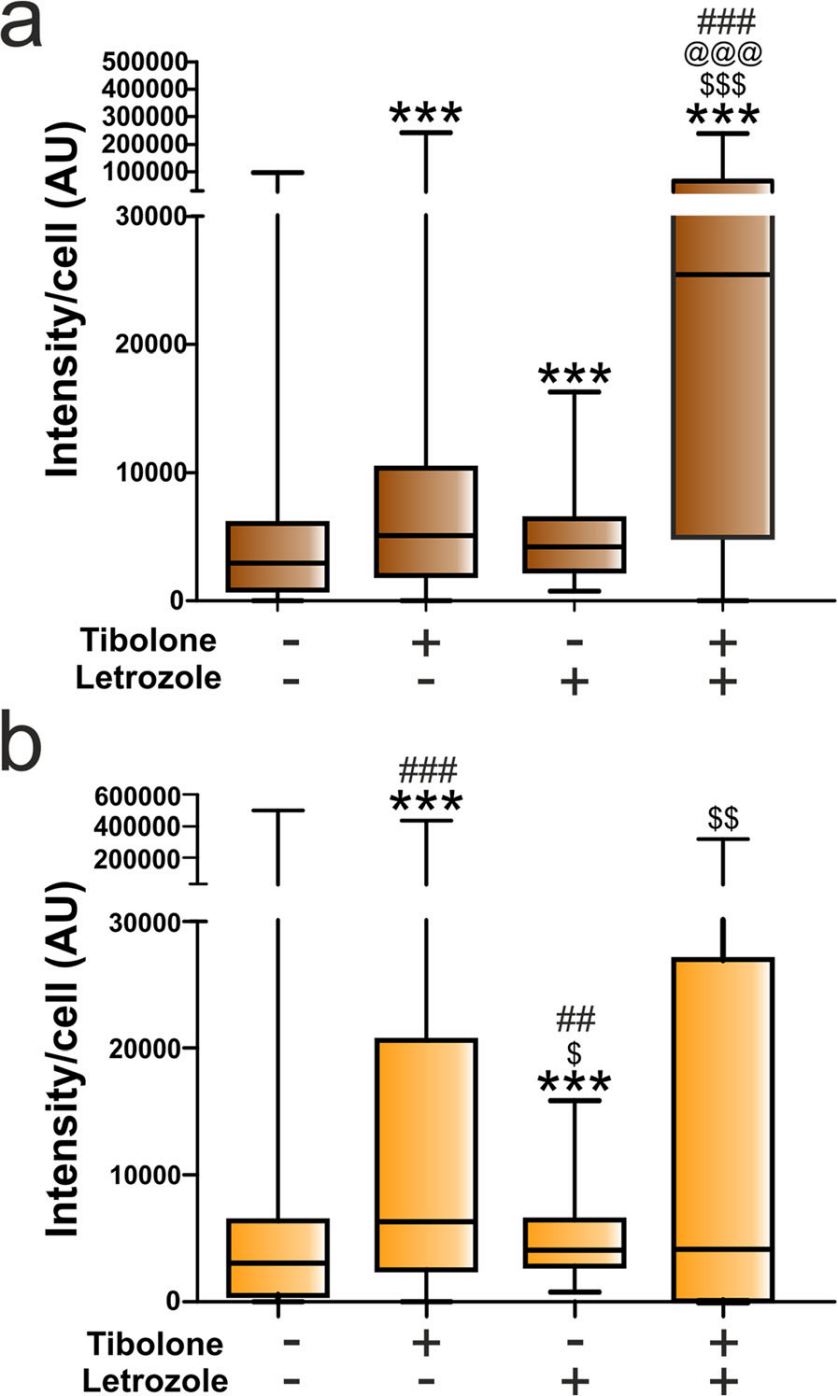
Quantitative analysis of the effect of tibolone and the aromatase inhibitor letrozole on the phagocytosis of brain-derived cellular debris. Cy3 fluorescence intensity per cell in male (**a**) and female (**b**) astrocytes treated for 39 h with tibolone, tibolone and/or letrozole, or control medium. Cells were then incubated for 1 h with Cy3-conjugated brain-derived cellular debris. Data are presented as median ± ranges. Significant differences: ***p < 0.001 versus the control group of the same sex, ^$$$^*p* < 0.001, ^$$^*p* < 0.01, ^$^*p* < 0.05 versus the tibolone group of the same sex, ^@@@^*p* < 0.001 versus the letrozole group of the same sex, ^###^*p* < 0.001, ^##^*p* < 0.01 versus the same experimental group of the other sex, being annotated in the group with higher median

The incubation of the cultures with letrozole resulted in a significant increase in the internalization of Cy3 in both male and female astrocytes (Fig. 6), with the effect being more pronounced in female cells. The increased internalization of Cy3 in the astrocytes incubated with letrozole suggests that endogenous estradiol reduces phagocytosis, or, alternatively, it may be interpreted as the result of the stimulation of phagocytosis by endogenous testosterone accumulated in the cultures as a consequence of the inhibition of aromatase [47]. In addition, letrozole enhanced the stimulatory effect of tibolone on Cy3 labeling of male astrocytes (Fig. 6a) but blocked the effect of tibolone in female astrocytes (Fig. 6b), reducing significantly the proportion on non-phagocytic female cells (from 3.69% in tibolone-treated cells to 15.32% in cells treated with tibolone and letrozole; *p* = 0.0052). This suggests that the balance in the endogenous levels of testosterone and estradiol had different outcomes for the effects of tibolone on male and female cells.

### Sex differences in the effect of tibolone on phagocytosis in astrocytes stimulated with LPS

**Fig. 7:**
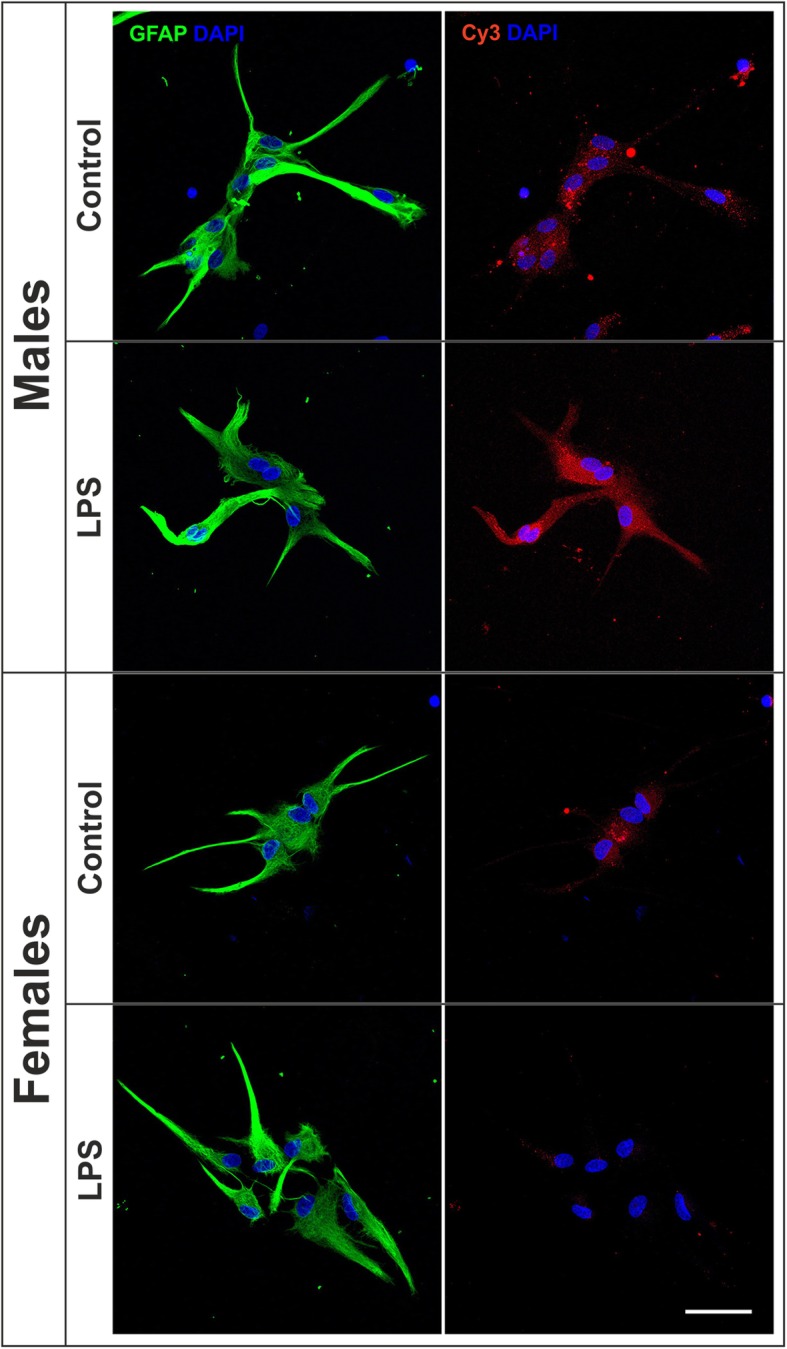
Effect of LPS on the phagocytosis of brain-derived cellular debris. Representative confocal images of male and female astrocyte cultures treated for 24 h with LPS or control medium and then incubated for 1 h with Cy3-conjugated brain-derived cellular debris (red) and immunostained for GFAP (green). Cell nuclei were stained with DAPI. Scale bar 50 μm

**Fig. 8:**
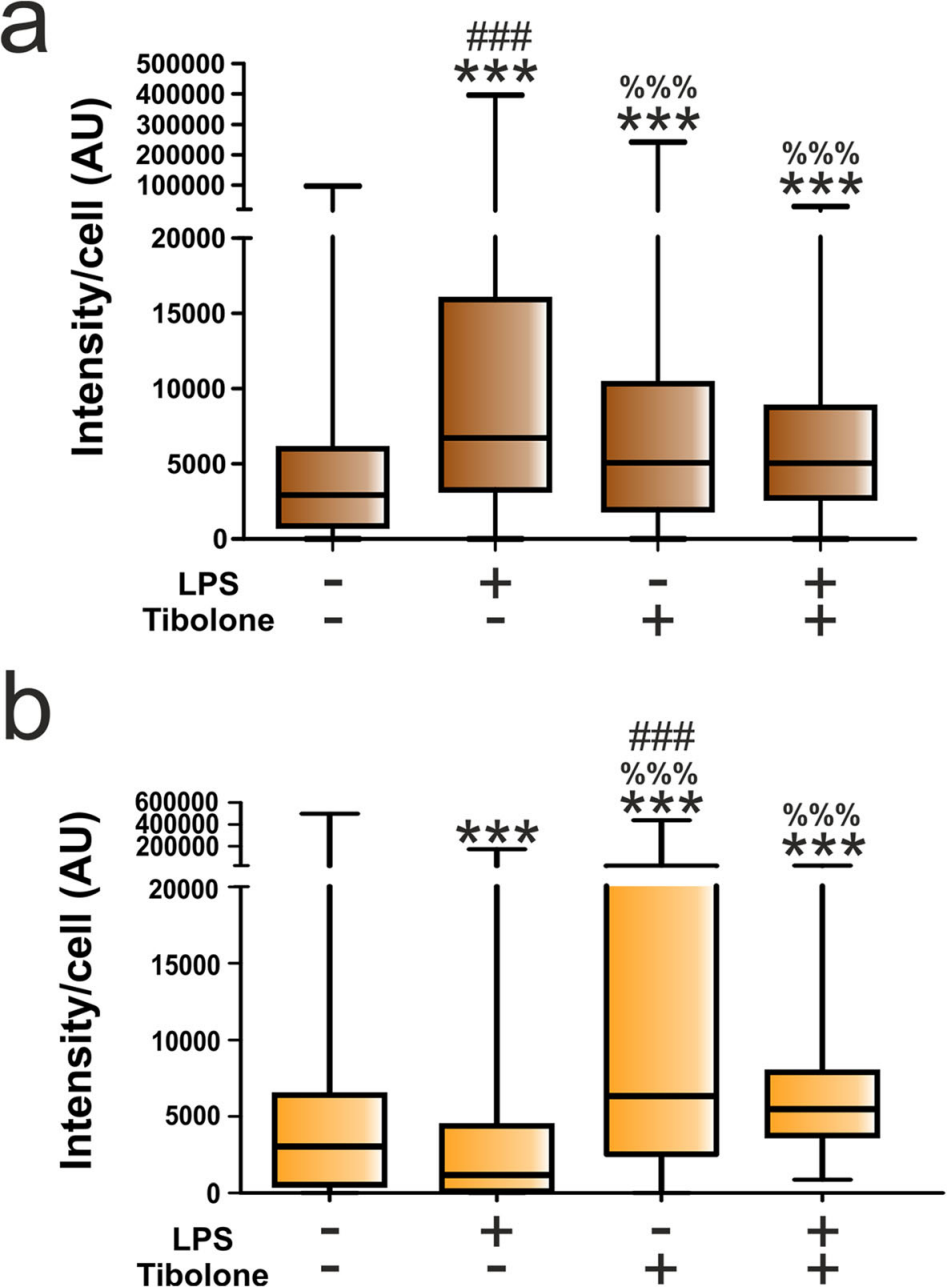
**a**, **b** Quantitative analysis of the effect of LPS and tibolone on the phagocytosis of brain-derived cellular debris. Cy3 fluorescence intensity per cell in male and female astrocytes treated for 39 h with tibolone or control medium and the last 24 h with LPS and then incubated for 1 h with Cy3-conjugated brain-derived cellular debris. Data are presented as median ± ranges. Significant differences: ***p < 0.001 versus the control group of the same sex, ^%%%^*p* < 0.001 versus the LPS group of the same sex, ^###^*p* < 0.001 versus the same group of the other sex, being annotated in the group with higher median

In the next experiment, we assessed the effect of tibolone on astrocyte phagocytosis under an immune challenge. Thus, astrocytes were treated with tibolone for 15 h and then with LPS for an additional 24 h. A significant sex difference was observed in Cy3 internalization of astrocytes after LPS stimulation. Thus, LPS significantly stimulated Cy3 labeling in male astrocytes, compared to basal conditions (Figs. 7 and 8a). In contrast, LPS exerted a significant inhibitory effect on Cy3 labeling of female astrocytes (Figs. 7 and 8b), increasing the proportion of non-phagocytic female cells from 2.87% in basal conditions to 10.24% after LPS treatment (*p* = 0.033).

Compared to control cells, tibolone stimulated phagocytosis in male and female astrocytes, both under basal conditions and after LPS stimulation. However, tibolone modulated the effect of LPS in both male (Fig. 8a) and female (Fig. 8b) cells, reducing phagocytosis in LPS-stimulated male astrocytes and increasing phagocytosis in LPS-stimulated female cells to the levels observed after the treatment with tibolone alone.

### Aromatase inhibition enhances the stimulatory effect of tibolone on phagocytosis in LPS-stimulated astrocytes of both sexes

**Fig. 9:**
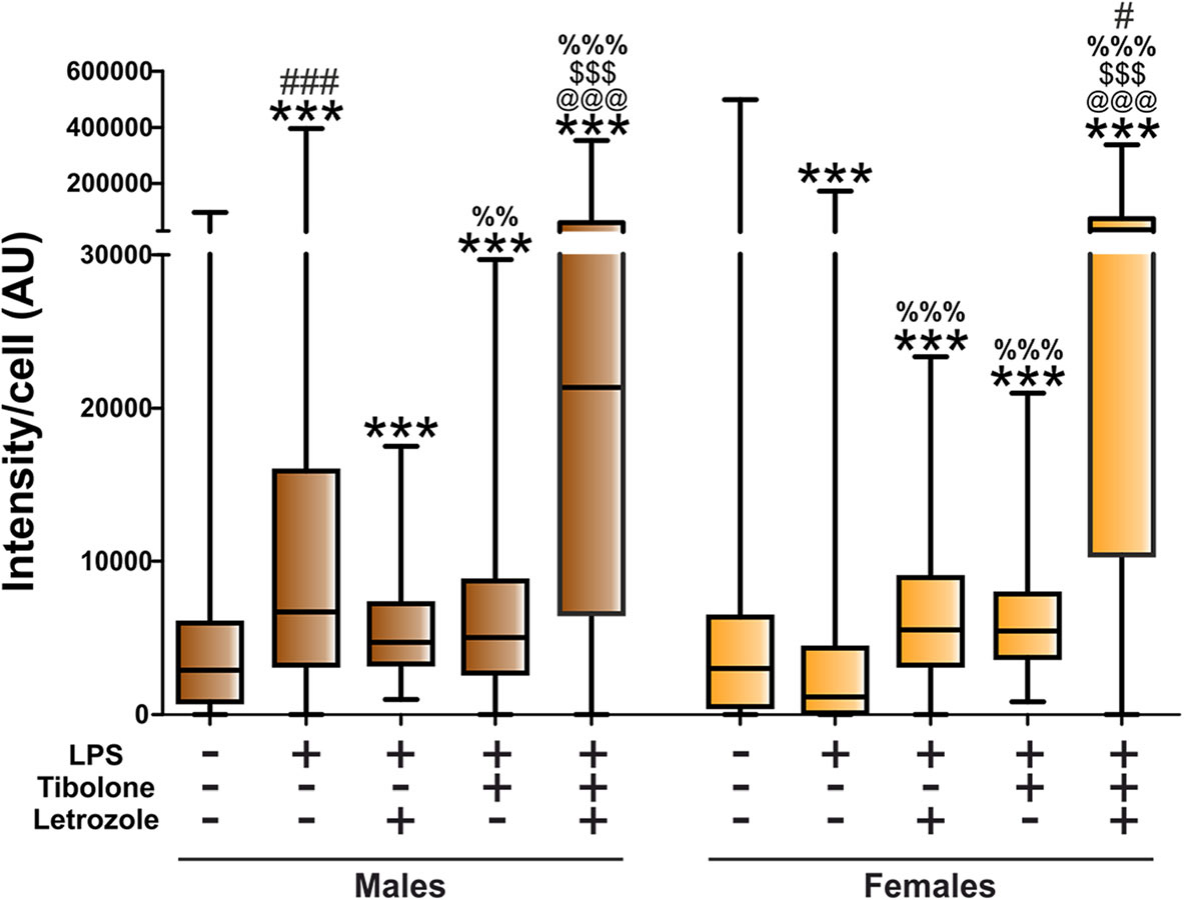
Quantitative analysis of the effect of letrozole, LPS, and tibolone on the phagocytosis of brain-derived cellular debris. Cy3 fluorescence intensity per cell in male and female astrocytes treated for 39 h with letrozole, tibolone, or control medium and 24 h with LPS and then incubated for 1 h with Cy3-conjugated brain-derived cellular debris. Data are presented as median ± ranges. Significant differences: ***p < 0.001 versus the control group of the same sex, ^%%%^*p* < 0.001; ^%%^*p* < 0.01 versus the LPS group of the same sex, ^$$$^*p* < 0.001 versus the LPS+letrozole group of the same sex, ^@@@^ *p* < 0.001 versus the LPS+tibolone group of the same sex, ^###^*p* < 0.001, ^#^*p* < 0.05 versus the same group of the other sex, being annotated in the group with higher median

In the following experiment, we studied the effects of LPS and tibolone in the presence of letrozole (Fig. 9). In male astrocytes, the incubation with letrozole significantly decreased the stimulatory effect of LPS on Cy3 internalization to a level that, however, was still higher than in control cultures. In contrast, letrozole significantly increased Cy3 labeling in LPS-treated female astrocytes over the control values and over the values of the cultures treated with LPS alone (Fig. 9). Under these conditions, the addition of tibolone resulted in a striking increase in Cy3 labeling in both sexes (Fig. 9). Thus, Cy3 labeling in the cultures treated with letrozole, LPS, and tibolone reached significantly higher values than in the cultures treated with LPS alone, LPS and tibolone, LPS and letrozole, or control medium (Fig. 9). In addition, Cy3 labeling was significantly higher in female than in male astrocytes after the treatment with letrozole, LPS, and tibolone (Fig. 9).

## Discussion

Recent evidence indicates that the phagocytic activity of astrocytes is involved in CNS remodeling under physiological and pathological conditions [24, 27, 28, 30, 48]. In the present study, we have confirmed the results of previous reports showing that astrocyte phagocytic activity can be assessed in monolayer cultures [49–51]. In addition, we show that tibolone stimulates the phagocytosis of brain-derived cellular debris by male and female astrocytes, both under basal conditions and after stimulation with LPS.

Our findings indicate that astrocytes have a similar basal phagocytic activity in both sexes, in contrast to what it has been reported for microglia and peripheral immune cells, where females have higher phagocytosis levels compared to males [52–54]. Tibolone stimulated phagocytosis in male and female astrocytes, with a significantly higher effect in female cells. The bigger effect of tibolone on female astrocytes may be due to sex differences in tibolone signaling. It is important to mention that previous studies on the effects of tibolone in the CNS have been mostly conducted in women or in female animals [2]. Thus, the mechanisms of tibolone action in male neural cells remain unexplored.

Previous studies have shown that tibolone exerts different actions in astrocyte cell lines by the activation of ERs, mainly through ERβ [14, 15]. In agreement with these previous reports, we observed that PHTPP, an ERβ antagonist, blocked the action of tibolone on phagocytosis in female astrocytes. In contrast, none of the tested ER antagonists were able to modify the effect of tibolone on phagocytosis in male astrocytes, suggesting that the action of the steroid in the cells of this sex either requires the combined activation of several ERs or is mediated by other receptors. Therefore, we also studied the effect of flutamide, an antagonist of the androgen receptor. In female astrocytes, flutamide blocked the effect of tibolone on phagocytosis, indicating that in astrocytes of this sex, tibolone requires both the ERβ and the androgen receptor to regulate phagocytosis. However, flutamide failed to block the effect of tibolone on male astrocytes. This indicates that the mechanism of action of tibolone to regulate phagocytosis is different in male and female astrocytes. Further investigation is therefore needed to determine the receptors involved in the regulation by tibolone of male astrocyte phagocytosis. Possible candidates are progesterone receptors, which may be activated by tibolone metabolites and they are also expressed in astrocytes [55]. Potential sex differences in progesterone receptor expression in astrocytes [56] could also affect estrogenic signaling in these cells, since progesterone modulates estrogen receptor activity [57]. However, the analysis of these receptors is problematic, given their variety of molecular structures and pharmacological characteristics [58, 59].

In addition to sex differences in tibolone signaling in astrocytes, another factor that may affect the outcome of tibolone treatment in male and female cells is the possible interference of the endogenous sex steroids with the effects of tibolone. Interestingly, aromatase inhibition with letrozole under basal conditions stimulated the effect of tibolone on phagocytosis in male astrocytes but inhibited the effect of tibolone in female cells to control levels. The different effects of aromatase inhibition in the phagocytic response of male and female astrocytes to tibolone suggests that endogenous steroids produced by glial cells may contribute to the generation of sex differences in the regulation of phagocytosis by this synthetic steroid under basal conditions. This is probably also the case under inflammatory conditions, since aromatase inhibition also affected phagocytosis in the cultures treated with LPS, as discussed below.

LPS induces an inflammatory response in glial cells [60] and previous studies have shown that it increases phagocytosis in astrocytes derived from the forebrain of unsexed newborn rats [49]. Our results indicate that the effect of LPS on phagocytosis is different in male and female cells. Thus, the phagocytosis of brain-derived cellular debris was stimulated in male astrocytes but inhibited in female cells. This sexually differentiated response is in agreement with the induction by LPS of sex differences in the gene expression of astrocytes [34, 39]. In addition, sex differences in morphology and gene expression have been detected in astrocytes under physiological conditions and in response to different pathological insults [35, 38]. Previous studies have shown that sex differences in astrocytes depend on organizational and activational actions of testosterone [37, 61]. Our study extends these previous observations of sex differences in gene expression to a functional activity, such as phagocytosis, that is activated in astrocytes in response to brain damage [28–30]. This difference in the phagocytic activity of male and female astrocytes after stimulation by an inflammatory challenge may contribute to the sex differences observed in the pathological manifestation of neurodegenerative diseases [62–67].

Our findings indicate that an inflammatory change may also alter the phagocytic response of male and female astrocytes to tibolone. It is important to consider that steroid signaling in astrocytes is upregulated under inflammatory conditions [68]. Therefore, under these circumstances, the response of astrocytes to tibolone may be altered. Indeed, compared to control conditions, tibolone increased phagocytosis after LPS stimulation in male and female cells. However, tibolone exerted a sex-specific modulation of the effect of LPS, decreasing phagocytosis in males and increasing phagocytosis in females, compared to the effect of LPS alone. Since tibolone is known to trigger a variety of cellular protective responses in astrocytes [12–14, 16], the different effects of this steroid after LPS stimulation may represent a sex-specific homeostatic regulation of phagocytosis, increasing or decreasing this process to counteract the effect of the pro-inflammatory stimulus.

As observed for basal conditions, astrocyte phagocytosis after an inflammatory stimulus was also affected by the inhibition of aromatase activity with letrozole. Thus, letrozole prevented the sexually differentiated response of astrocytes to LPS and aromatase inhibition strongly enhanced phagocytosis in the cultures stimulated with LPS and treated with tibolone. These treatments result in a higher phagocytosis in female astrocytes compared to male cells. It is known that aromatase expression is upregulated in astrocytes under inflammatory conditions [69, 70], but with significant differences in males and females [71]. Therefore sex-specific modifications in the metabolism of endogenous testosterone to estradiol under these circumstances may cause different effects of letrozole in males and females and contribute to the observed changes in phagocytosis. In summary, our findings suggest that the balance of endogenous androgens and estrogens influence the action of tibolone on phagocytosis of male and female astrocytes, both under basal conditions and upon inflammatory stimulation.

## Conclusions

**Fig. 10:**
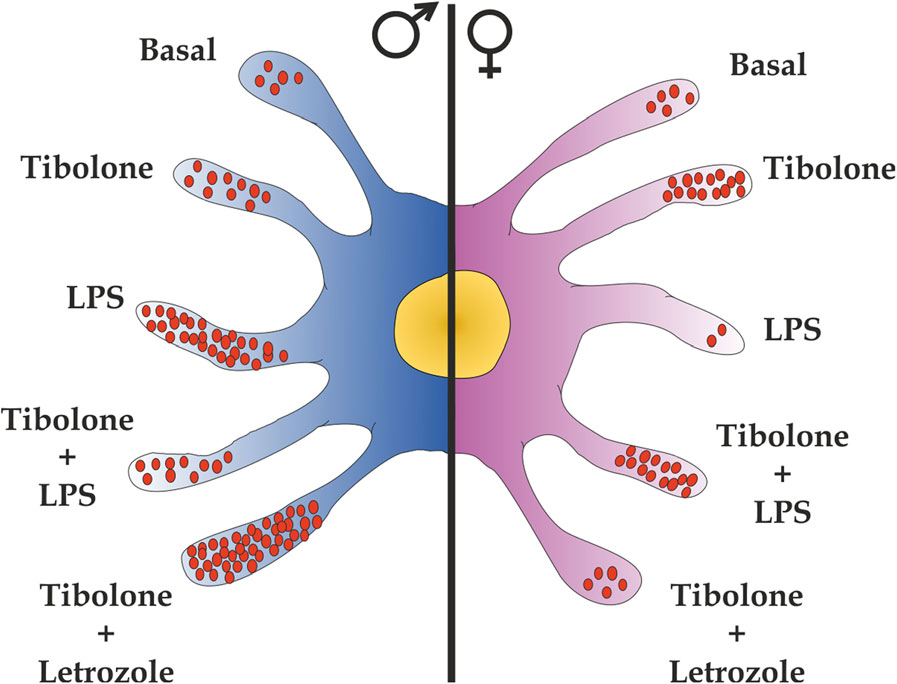
Summary of the effect of tibolone in male and female astrocytes. Under basal conditions, both male and female astrocytes have a similar phagocytic activity. Tibolone increases phagocytosis in astrocytes of both sexes, with a higher effect in females. In female astrocytes, the effect of tibolone was blocked with the inhibition of local estradiol synthesis using letrozole. In contrast, in male astrocytes, the action of tibolone is increased by the inhibition of local estradiol synthesis. Under an inflammatory challenge (LPS), phagocytosis is increased in male astrocytes and decreased in female cells. Under these conditions, tibolone restores phagocytosis to basal tibolone levels in both sexes

The findings of the present study outlined in Fig. 10 indicate that tibolone enhances phagocytosis of brain-derived cellular debris by primary astrocytes of both sexes, having a bigger effect in female cells. This effect of tibolone involves different molecular mechanisms in male and female astrocytes, with ERβ and androgen receptor being involved in the effect of the steroid in female cells. In addition, tibolone regulates in a sex-specific manner the phagocytosis of cellular debris by astrocytes after an inflammatory challenge but stimulates phagocytosis in male and female cells under inflammatory conditions when the activity of aromatase is inhibited, indicating that the response of astrocytes to the synthetic steroid is modulated by the endogenous balance of testosterone and estradiol. Since phagocytosis in astrocytes is activated under pathological conditions [28–30], the control of astrocyte phagocytosis by tibolone may have relevant implications for the neuroprotective actions of this synthetic steroid.

## Data Availability

All data generated or analyzed during this study are included in this published article (and its supplementary information files).

## Abbreviations

CNS: Central nervous system
ERs: Estrogen receptors
Flut: Flutamide
GFAP: Anti-glial fibrillary acidic protein
LPS: Lipopolysaccharide
